# Comparison of BSGI, MRI, mammography, and ultrasound for the diagnosis of breast lesions and their correlations with specific molecular subtypes in Chinese women

**DOI:** 10.1186/s12880-020-00497-w

**Published:** 2020-08-15

**Authors:** Hongbiao Liu, Hongwei Zhan, Da Sun, Ying Zhang

**Affiliations:** Department of Nuclear Medicine, The Second Affiliated Hospital, Zhejiang University School of Medicine, 88 Jiefang Road, Hangzhou, China

**Keywords:** BSGI, Mammography, Ultrasound, MRI, Scintigraphy

## Abstract

**Background:**

Breast cancer is a leading cause of cancer in females, and is the second leading cancer-related cause of death in this group. Early diagnosis is essential to breast cancer to be effectively treated, and ultrasound, mammography, and magnetic resonance imaging (MRI) represent three key technologies that are utilized for the diagnosis of breast lesions. Breast-specific gamma imaging (BSGI) is an approach to molecular breast imaging that allows for high-resolution radio-imaging that is not adversely impacted by breast tissue density. This study was therefore designed to assess the relative diagnostic efficacy of BSGI, MRI, mammography, and ultrasound in different molecular subtypes of breast cancer among Chinese women.

**Methods:**

Diagnostic findings from 390 patients that had undergone diagnosis and treatment in our breast surgery department were retrospectively reviewed. Patients had been diagnosed via BSGI, mammography, ultrasound, and MRI. The diagnostic efficacy of these different imaging modalities and their associated biological characteristics were compared in the present study.

**Results:**

A total of 229 of these 390 patients (58.7%) were diagnosed with malignant breast cancer, with the remaining 161 (41.3%) cases having been found to be benign. BSGI, MRI, mammography, and ultrasound yielded respective sensitivity values of 91.7, 92.5, 77.3, and 82.1%, while the respective specificity values for these imaging modalities were 80.7, 69.7, 74.5, and 70.8%. For lesions > 1 cm, BSGI offered a sensitivity of 92.5%. For mammographic breast density A, B, C, and D, BSGI offered a sensitivity of 93.3, 94.0, 91.5, and 89.3%, respectively. BSGI also yielded a significantly higher lesion-to-normal lesion ratio (LNR) for malignant lesions relative to benign lesions (2.76 ± 1.32 vs 1.46 ± 0.49).

**Conclusions:**

These findings confirm that BSGI is highly sensitive and is superior to mammography in the detection and diagnosis of ductal carcinomas in situ (DCIS). Such diagnostic efficacy can be further improved by using BSGI as an auxiliary modality to mammography and ultrasound, potentially improving the reliability of breast lesion diagnosis, thereby ensuring that patients receive rapid and effective treatment without the risk of misdiagnosis or unnecessary surgical treatment.

## Background

Breast cancer remains the third leading cause of cancer globally and the leading cancer-related cause of death in women, with 1.7 million new cases having been diagnosed in 2016 [1]. The exact etiological basis for breast cancer is complex and remains to be fully clarified. However, as the rapid diagnosis of early-stage disease is the most reliable means of achieving a positive prognosis, the identification of optimal diagnostic approaches for the analysis of breast lesions is essential. Mammography (MMG) remains the primary approach used for breast cancer screening and detection throughout the world, with subsequent ultrasound (US) being used to confirm cancer diagnosis, for image-guided breast biopsy and localization, for axillary assessment, and to follow-up on any abnormal magnetic resonance imaging (MRI). Breast MRI is the most sensitive approach to detecting and diagnosing breast cancer. Breast-specific gamma imaging (BSGI), also known as ^99m^Tc-sestamibi scintigraphy, is a high-resolution molecular breast radio-imaging approach that can also be used to precisely detect breast cancer in tissues of variable density. In the present manuscript, we compared the efficacy of BSGI, ultrasound, mammography, and MRI for the differential diagnosis of breast lesions in order to better understand the relative clinical value of BSGI as a diagnostic tool, and found that such diagnostic processes can be improved by using ^99m^Tc-sestamibi scintigraphy as an auxiliary modality to mammography and ultrasound, thereby potentially improving the reliability of breast lesion diagnosis.

## Methods

### General information

This retrospective analysis was approved by our Hospital ethics committee, with all patients having provided written informed consent. In total, data pertaining to 390 patients that underwent BSGI at the Hospital (Second Affiliated Hospital of Zhejiang University School of Medicine, Hangzhou, China) from January 2015–December 2018 were analyzed. All of these patients underwent subsequent pathological evaluation of analyzed breast lesions via either core needle biopsy or surgical excision, and all patients had undergone ultrasound, mammography, and BSGI before diagnosis. We revised patient medical records in order to assess key clinicopathological parameters in these patients including age, lesion location, tumor size, grade, and tumor histological type.

### Assessment of tumor pathology

The World Health Organization (WHO) classification system was used to define the pathological characteristics of breast tumors in patients in the present study. Estrogen and progesterone receptor (ER and PR, respectively) expression on these tumors was evaluated via immunohistochemistry (IHC), with tumors being considered ER- or PR-positive when at least 1% of tumor cells stained positive for these respective receptors. The American Society of Clinical Oncology/College of American Pathologists guidelines were used to define Her-2 status for analyzed tumors. No fluorescence in situ hybridization was conducted for borderline cases, and scores of both 1+ and 2+ were considered to be negative [2]. The expression of Ki-67 was assessed by evaluating the average percentage of cells with nuclear staining in 10 high-power microscope fields of view, with > 14% of cells staining positive being used as the threshold to define Ki-67 positivity [3]. Breast tumors were classified into four subtypes according to ER, PR, and Her-2 expression status as follows: Luminal A (ER^+^ and/or PR^+^, and Her-2^−^), Luminal B (ER^+^ and/or PR^+^ and Her-2^−^), Her-2 positive (ER^−^, PR^−^ and Her-2^+^) and triple-negative (ER^−^, PR^−^ and Her-2^−^).

### BSGI review

For BSGI, patients maintained a normal diet and underwent no specific preparation. They were then administered an antecubital vein (contralateral to the breast lesion) injection of 555-740 MBq (Shanghai GMS Pharmaceutical Co., Ltd) ^99m^Tc-sestamibi. Ten minutes later, BSGI was conducted, with patients remaining in a seated position and being imaged using a breast-specific gamma camera (Dilon 6800; Dilon Technologies Inc., USA) to acquire high-resolution bilateral craniocaudal (CC) and mediolateral oblique (MLO) images. Each image was acquired for roughly 5 min, with 100,000 counts/image being the defined minimal range [4, 5]. All BSGI findings were interpreted based upon operative guidelines published by the Society of Nuclear Medicine by two experienced physicians specializing in nuclear medicine. Positive BSGI tumors that were found to exhibit a lesion-to-normal lesion ratio (LNR) > 1.65 were considered to be highly suspicious.

### Ultrasound

Ultrasound was conducted with patients in the supine position (Philips Healthcare, Netherlands). Those lesions with a suspicious appearance upon ultrasound analysis, such as those exhibiting irregular solid hypoechoic nodules, a vertical to horizontal ratio of > 1, uneven edges, or punctate calcification were considered to be positive and to warrant biopsy or removal.

### Mammography

Mammography was conducted with an appropriate mammographic instrument (Hologic, USA) with patients in the erect position. Mammographic breast density was estimated visually based upon the American College of Radiology Breast Imaging-Reporting and Data System (BI-RADS) classification system as follows: density category 1 (dense A, glandular tissue ≤25%), density category 2 (dense B, glandular tissue, 25–50%), density category 3 (dense C, glandular tissue, 51–75%), or density category 4 (dense D, glandular tissue ≥75%). All mammography images were independently evaluated and interpreted by two radiologists according to these criteria. Disagreements were resolved through discussion and consensus.

### MRI

A 1.5 T system (Siemens, Germany) and a dedicated breast coil were used to conduct MRI, with patients in the prone position. All MRI images were interpreted according to the BI-RADS classification by two radiologists. Disagreements were resolved through discussion and consensus.

## Statistical analysis

Sensitivity, specificity, and positive and negative predictive values for BSGI, ultrasound, mammography, and MRI were determined. In addition, χ^2^ tests were used to compare the relative efficiencies of these four imaging modalities and associated indicators. The correlation between the LNR of lesions measured by malignant tumors and benign lesions and by pathology was investigated via a Pearson nonparametric correlation analysis. All statistical testing was conducted using SPSS v.22 with a significance threshold of *P* < 0.05.

## Results

### Patient characteristics

In total, 390 female patients that had undergone BSGI, ultrasound, and mammography were included in the present study, of whom 235 had also undergone MRI.

### Pathologic results

Of these 390 patients, 229 (58.7%) were ultimately diagnosed with malignant tumors. These patients had an average age of 49.7 years (range:2 3–89). Detected malignancies included invasive ductal carcinoma (IDC, *n* = 186), ductal carcinomas in situ (DCIS, n = 18), breast Paget’s disease (*n* = 4), tubular carcinoma (n = 4), invasive lobular carcinoma (n = 4), carcinoma with apocrine differentiation (*n* = 3), malignant phyllodes tumor (*n* = 2), mucinous carcinoma (*n* = 1), carcinoma with signet-ring-cell differentiation (*n* = 1), invasive papillary carcinoma (*n* = 1), diffuse large B-cell lymphoma (*n* = 1), carcinoma with neuroendocrine differentiation (*n* = 1), mixed metaplastic carcinoma (*n* = 1), adenoid cystic carcinoma (*n* = 1) and invasive micropapillary carcinoma (*n* = 1). The other 161 patients in the present study (41.3%) were diagnosed with benign lesions. These patients had an average age of 45.3 years (range: 19–74). Detected benign lesions included fibroadenomas (*n* = 49), adenosis (*n* = 47), usual ductal hyperplasia (*n* = 26), intraductal papillomas (*n* = 21), breast cysts (*n* = 12), and chronic inflammation (*n* = 6).

### BSGI, ultrasound, mammography, and MRI diagnostic efficacy

**Fig. 1:**
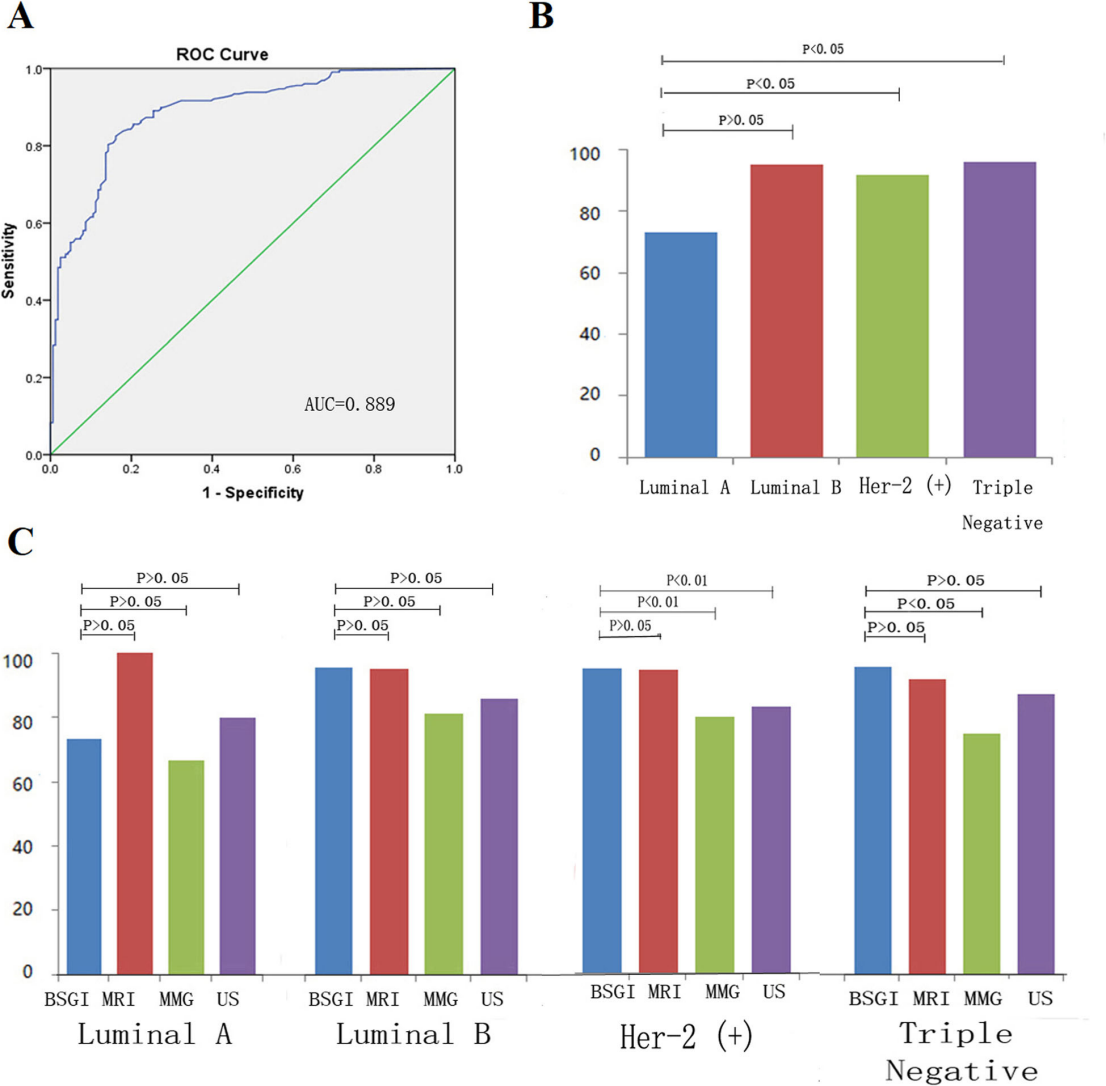
Determination of an optimal LNR cut-off value for breast cancer detection. **a**. Determination of an optimal LNR cut-off value for breast cancer detection via a ROC curve approach. **b**. BSGI sensitivity for the evaluation of different breast cancer molecular subtypes. **c**. Sensitivity of BSGI, MRI, mammography, and ultrasound for the evaluation of different breast cancer molecular subtypes

**Fig. 2:**
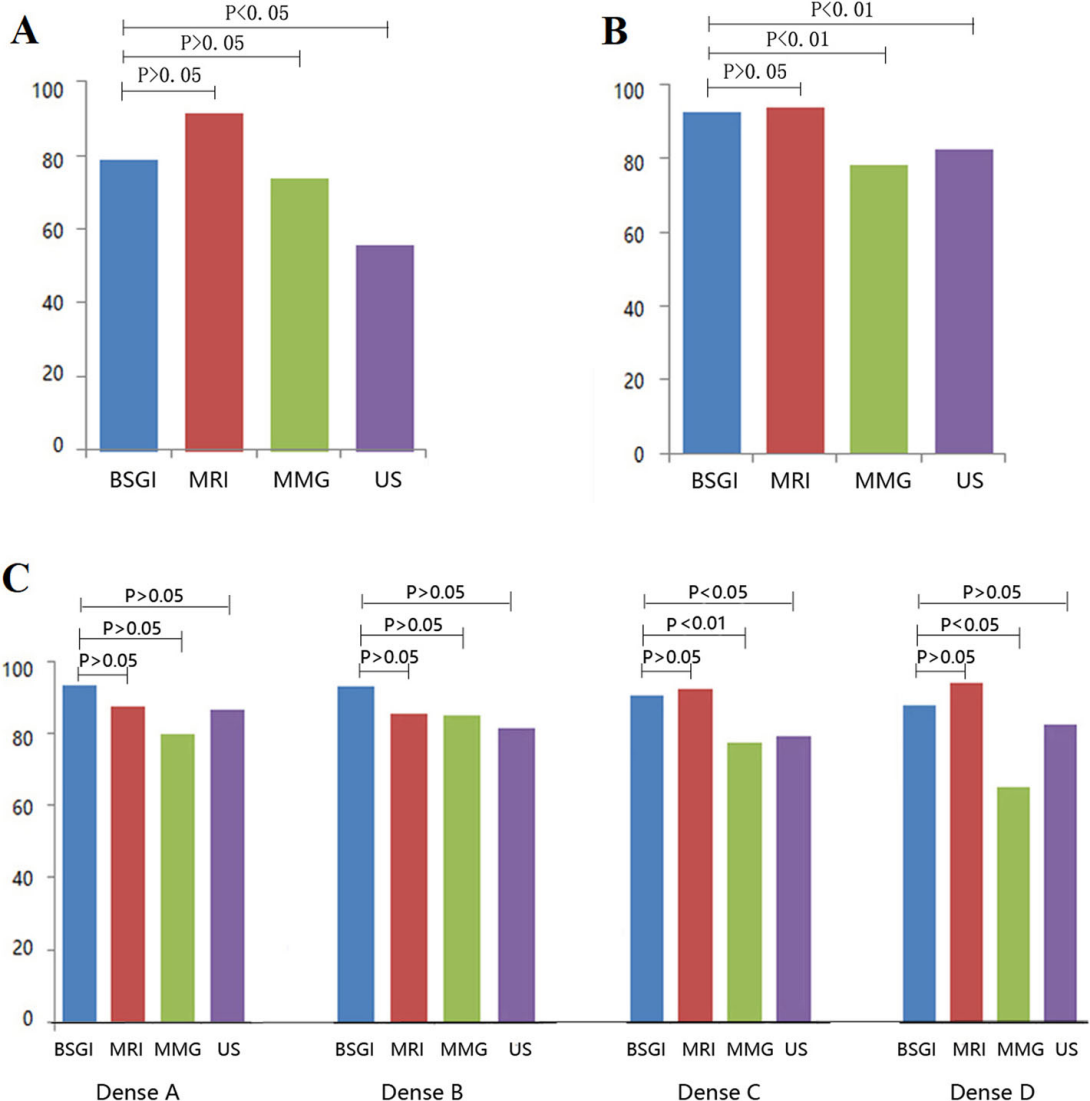
Sensitivity of BSGI, ultrasound, mammography, and MRI in different types of breast cancer. **a**. BSGI, MRI, mammography, and ultrasound sensitivity for the detection of DCIS. **b**. Sensitivity of BSGI, MRI, mammography, and ultrasound for the evaluation of lesions > 1 cm in size. **c**. Sensitivity of BSGI, MRI, mammography, and ultrasound for the diagnosis of breast cancer in breast tissues of differing densities

In this patient cohort, BSGI, MRI, mammography, and ultrasound achieved sensitivity values of 91.7, 92.5, 77.3, and 82.1%, respectively, for the diagnosis of malignant lesions. While BSGI had an overall sensitivity of 91.7%, this value varied based upon tumor type and was 93.3% for IDC, 78.9% for DCIS, 100% for breast Paget’s disease, 100% for invasive lobular carcinoma, and 75.0% for tubular carcinoma. BSGI achieved a specificity of 80.7%, while for ultrasound the specificity was just 70.8% (χ^2^ = 4.33,*P* < 0.05) (Fig. 2a). While BSGI sensitivity was comparable for Luminal-A and Luminal-B tumors, its sensitivity for Her-2-positive and triple-negative tumors was superior to that for Luminal-A tumors (Fig. 1b). We found lesion malignancy and the LNR ratio to be significantly correlated with one another, with respective LNR ratio values of 2.76 ± 1.32 and 1.46 ± 0.49 in patients diagnosed with malignant and benign lesions, respectively (*t* = 31.56, *P*<0.01; *r* = 0.518, *P* < 0.01). Based upon these results, an LNR ratio cut-off value of 1.65 was selected to optimize sensitivity and specificity (Sensitivity: 86.0%, Specificity: 78.6%) (Fig. 1a). BSGI achieved superior sensitivity to ultrasound for the detection of Her-2-positive tumors (Fig. 1c). For further details regarding these sensitivity and specificity findings, see Table 1.

**Table 1:** Diagnostic efficacy of BSGI, ultrasound, mammography, and MRI

Approach	Sensitivity	Specificity	PPV	NPV
BSGI	91.7 (210/229)*a	80.7 (130/161)*b	87.1 (210/241)*c	87.2 (130/149)*d
MRI	92.5 (147/159)	69.7 (53/76)	86.5 (147/170)	81.5 (53/65)
Mammography	77.3 (177/229)	74.5 (120/161)	81.2 (177/218)	69.8 (120/172)
Ultrasound	82.1 (188/229)	70.8 (114/161)	80.0 (188/235)	73.5 (114/155)

(*PPV* positive predictive value, *NPV* negative predictive value)

*a) BSGI to Mammography, χ^2^ = 18.15, *P* < 0.01, to Ultrasound, χ^2^ = 9.28,*P* < 0.01

*b) BSGI to Ultrasound, χ^2^ = 4.33,*P* < 0.05. *c) BSGI to Ultrasound, χ^2^ = 4.42, *P* < 0.05

*d) BSGI to Mammography, χ^2^ = 14.16,*P* < 0.01, to Ultrasound, χ^2^ = 9.0,*P* < 0.01

BSGI achieved sensitivity for DCIS that was markedly increased relative to ultrasound (78.9% vs. 55.6%, Fig. 2a). Of the analyzed lesions, those that were malignant ranged from 3 to 74 mm in size, with 17 of them being under 10 mm in size. BSGI achieved sensitivity comparable to that of MRI (92.5% vs. 93.9%) for lesions > 1 cm in size, while remaining superior to mammography and ultrasound (Fig. 2b). For these analyzes, the BI-RADS classifications were utilized as a standard means of reporting density findings in mammography analyses. Dense breast tissue was observed in 71.6% (164/229) patients that were ultimately diagnosed with malignancies. In those patients with dense D-type breast tissue, BSGI achieved significantly higher sensitivity than did mammography (89.3% vs. 66.1%, χ^2^ = 8.70,*P* < 0.05). Our results indicated that mammography sensitivity is sensitive to breast density, with these values falling from 86.0–66.1% with rising density. BSGI achieved respective sensitivity values of 93.3, 94.0, 91.5, and 89.3% when used to image breast tissue in the A, B, C, and D BI-RADS density categories (Fig. 2c).

**Fig. 3:**
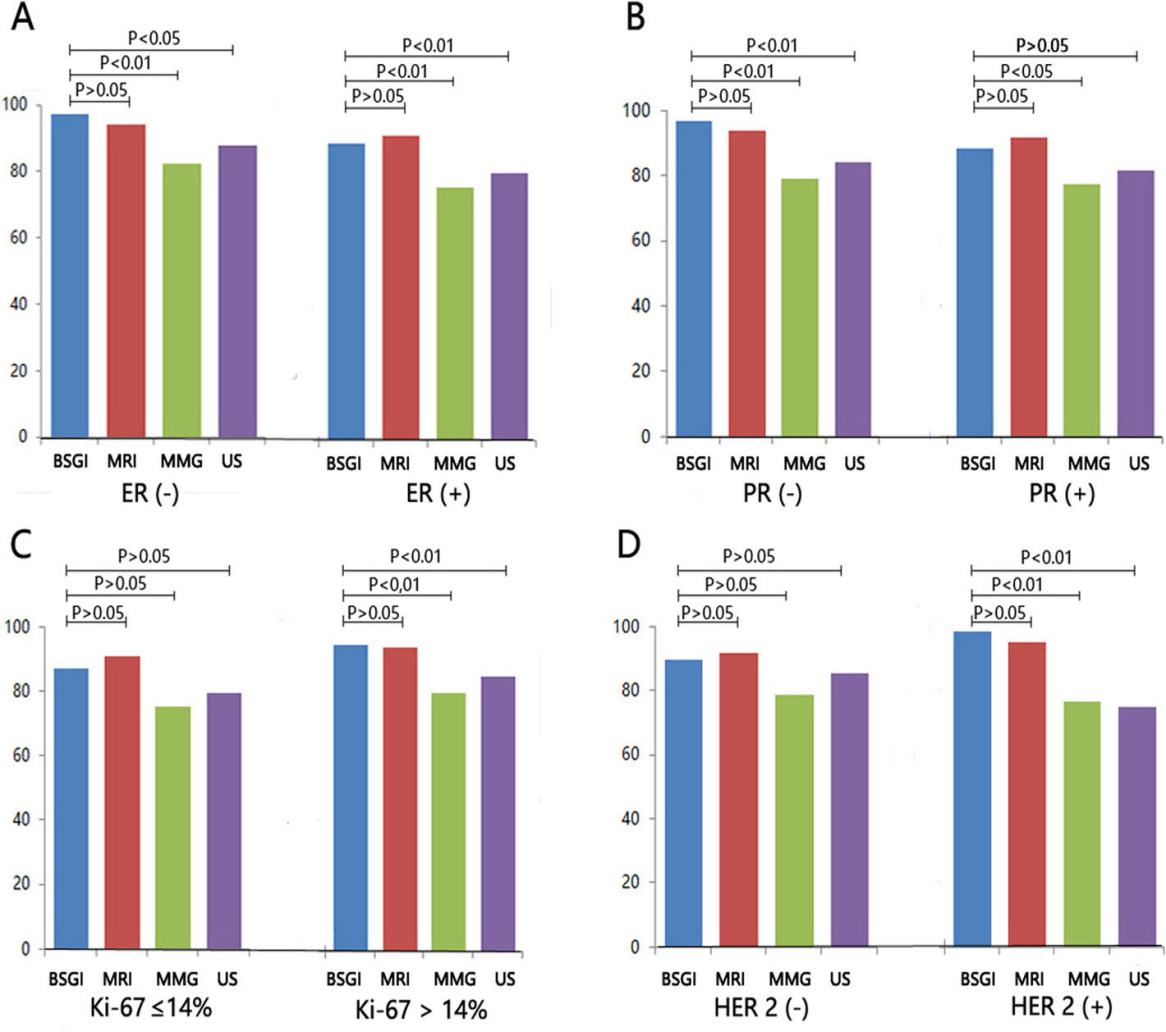
BSGI, ultrasound, mammography, and MRI sensitivity in different breast cancer types

IHC staining results were additionally analyzed for all patients included in the present study, of whom 156 (68.1%), 133(58.1%), and 57 (24.9%) were ER-positive, PR-positive, and Her-2-positive, respectively. In addition, tumors from 46 patients (20.1%) were found to exhibit a high Ki-67 index. Based on these molecular features, 15 (6.5%), 21 (9.2%), 169 (73.8%), and 24 (10.5%) tumors were classified as being of the Luminal-A, Luminal-B, Her-2-positive, and triple-negative subtypes. No significant differences in BSGI diagnostic utility were observed as a function of molecular subtype or ER, PR, Her-2, or Ki67 status. In addition, no significant differences were observed between BSGI and MRI with respect to diagnostic efficacy as a function of ER, PR, Her-2, or Ki67 status in the present patient cohort (Fig. 3).

### False-positive and false-negative BSGI findings

In total, 31 and 19 respective false-positive and false-negative BSGI findings were identified in the present patient cohort (Table 2).

**Table 2:** Analysis of BSGI false-positive and false-negative in the diagnosis of breast cancer

False-positive		False-negative	
Classification	Number	Classification	Number
Fibroadenoma	9	Invasive ductal carcinomas	13
Benign epithelial proliferation	5	Ductal carcinomas in situ	4
Chronic inflammation	5	Tubular carcinoma	1
Breast Cyst	5	Carcinoma with apocrine differentiation	1
Intraductal proliferative lesions	4		
Intraductal papilloma	3		

## Discussion

**Fig. 4:**
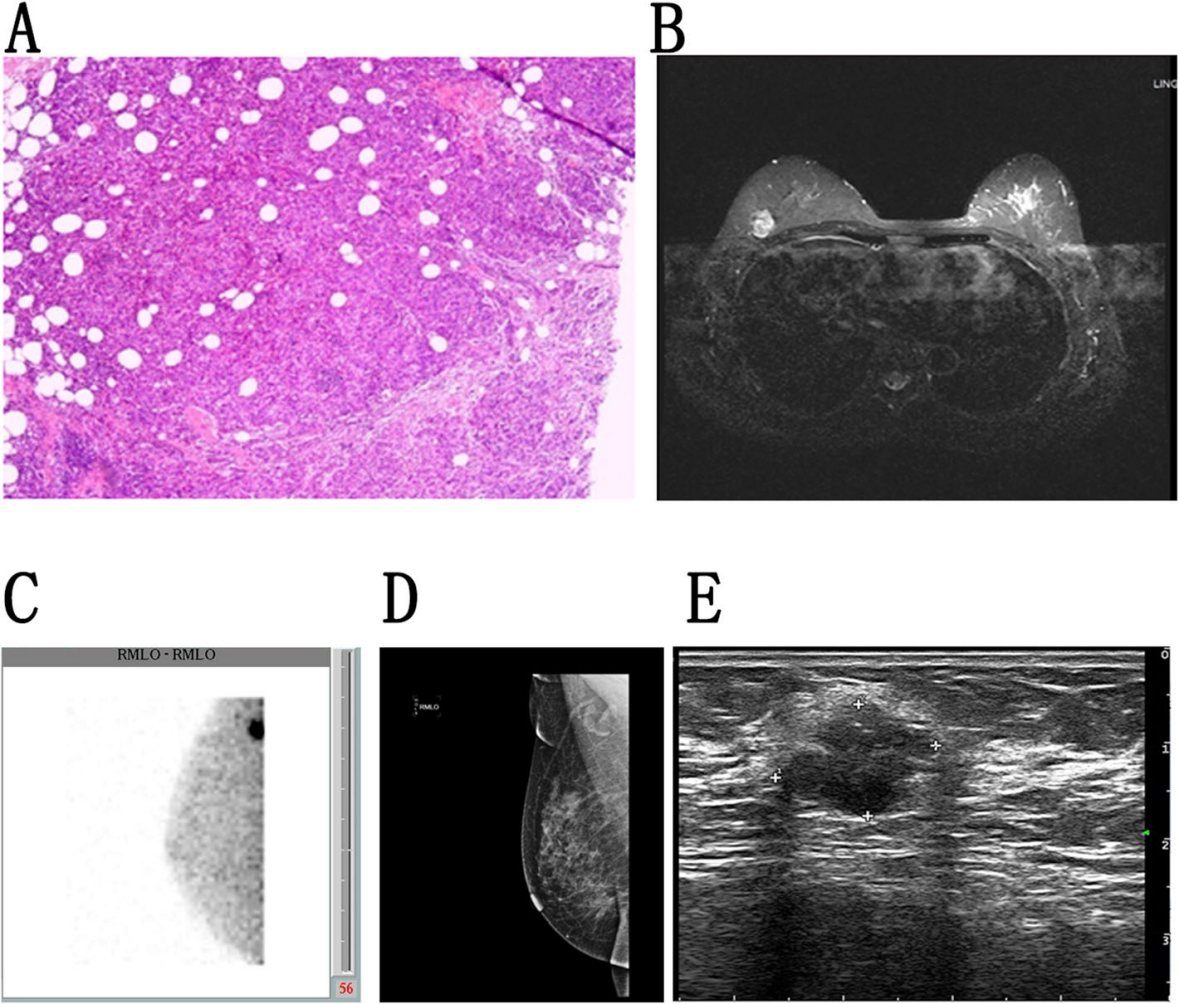
Breast scans from a woman. **a**. Pathological findings demonstrating invasive ductal carcinomas. **b**. MRI analysis revealed a nodular mass (BI-RADS 3) in the right breast that was found to be benign (1.2 × 1.4 cm). **c**. BSGI (RMLO) revealed focal enhanced radiotracer uptake in the right breast that was found to be malignant, LNR = 3.03. **d**. Mammography (RMLO) revealed breasts of density class C and the presence of a nodular-like (BI-RADS 3) structure in the upper outer quadrant area. **e**. Ultrasound detected a lobulated hypoechoic lesion (BI-RADS 4A) in the right breast, 1.5 × 1.2 cm

This is the first study to our knowledge to have compared the relative diagnostic efficacy of BSGI, mammography, ultrasound, and MRI as a means of differentiating between benign and malignant breast lesions as a function of tumor molecular subtype among Chinese women. Overall, we found that BSGI was an effective approach to diagnosing suspicious lesions. Mammography-based screening is generally the first-line approach to detecting breast cancer in patients with no overt signs of disease, and as such, this approach has seen widespread clinical implementation in recent decades. However, mammography is sensitive to breast density and to the presence of scar tissue, with increasing breast density being associated with reductions in sensitivity from 85 to 68%. Breast density is also a risk factor for breast cancer development [6–8], and upwards of 75% of Chinese women have heterogeneously or extremely dense breasts. As such, mammography-based screening approaches are of limited utility in these women, and alternative imaging modalities are thus required. BSGI is unaffected by breast density, scar tissue, structural deformities, or radiation therapy. In a retrospective analysis of 341 women that underwent BSGI prior to surgical tumor removal, Rechtman et al. [9] found BSGI to have an overall sensitivity of 95.4% for breast cancer detection, and they also determined that parenchymal breast density failed to impact such sensitivity. Consistent with these findings, in the present study we observed comparable BSGI sensitivity in dense A-D breast tissue. Chung et al. [10] previously conducted a retrospective analysis of 302 breast lesions in 266 women, and in so doing determined that BSGI was more specific than was adjunctive ultrasound US without any sensitivity loss, suggesting that adjunctive BSGI may be a valuable complementary imaging approach to detecting breast cancer in women with suspicious mammography findings. In our study, BSGI achieved the highest specificity (80.7%) of all four tested imaging modalities (Fig. 4).

Much like mammography, ultrasound is an anatomical approach to breast cancer diagnosis that is non-invasive, efficient, and convenient. As such, ultrasound is widely employed in clinical settings for the BI-RADS breast lesion classification. When women with palpable breast masses present for imaging and initial mammography findings are negative, breast cancer cannot generally be excluded. Ultrasound is thus commonly used as an adjunctive imaging modality in these women, particularly in those with dense breast tissue [11]. Tadwalkar et al. [12] retrospectively analyzed BSGI results in 129 women with invasive carcinoma, and found the efficiency of this diagnostic approach to be related to both tumor size and differentiation status. For lesions < 1 cm, BSGI exhibited sensitivity comparable to that of MRI, mammography, and ultrasound, whereas BSGI was superior to ultrasound and mammography when lesions were > 1 cm in size.

While physiological nuclear imaging approaches are commonly used to diagnose tumors, using auxiliary methods in addition to these physiology-based approaches can significantly improve rates of tumor detection. BSGI is a functional imaging modality that achieves improved sensitivity in patients with dense breasts and tumors < 1 cm in size relative to traditional planar scintigraphy [13]. Following injection into patients, the radioactive tracer ^99m^Tc-sestamibi can enter mitochondria. As numbers of mitochondria are generally correlated with cellular metabolic activity, highly metabolically active cancer cells take up ^99m^Tc-sestamibi more readily than do cells in the surrounding tissue. Indeed, tumor cells take up ^99m^Tc-sestamibi at rates ≥50% higher than do normal cells [14]. Kim et al. [15] conducted a retrospective study of 520 patients with suspected breast cancer that were evaluated via ^99m^Tc-sestamibi imaging, and found that lesions from patients diagnosed with malignant disease exhibited significantly higher LNR values than did those with benign disease (2.00 ± 1.88 vs. 0.60 ± 0.70). Choi et al. [16] similarly found LNR values to be higher in malignant breast lesions relative to benign lesions (2.2 ± 1.0 vs 1.6 ± 0.5). Consistent with these past results, we found that patients ultimately diagnosed with malignant lesions exhibited an LNR of 2.66 ± 1.32, with this value being significantly increased relative to that of patients with benign lesions (1.46 ± 0.49).

**Fig. 5:**
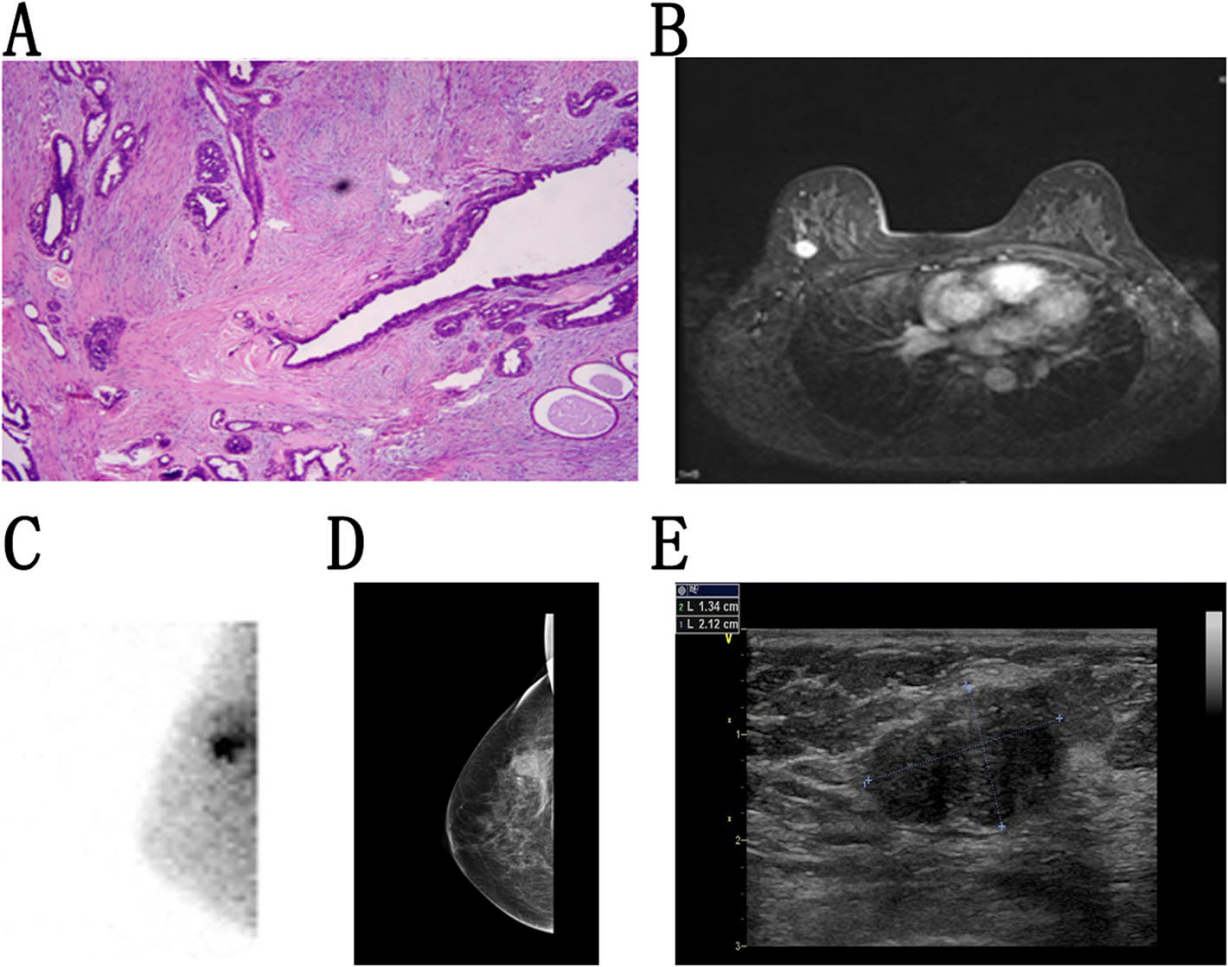
Breast images from a woman. **a**. Pathology identified a fibroadenoma. **b**. MRI results revealed a nodular mass (BI-RADS 4C) in the right breast,1.9 × 1.5 cm. **c**. BSGI revealed focally increased radiotracer uptake in the right breast that was consistent with malignancy, LNR = 2.49. **d**. Mammography demonstrated a high-density shadow (BI-RADS 4C) in the upper outer region of the right breast, 1.3 × 1.8 cm. **e**. Ultrasound revealed an elliptical hypoechoic mass (BI-RADS 4C) in the right breast, 1.3 × 2.1 cm

^99m^Tc-sestamibi is a non-specific agent when used for tumor imaging, and as such it can yield false-positive results when taken up by hyperplasic benign lesions, thus reducing its diagnostic performance. Fibrocystic breast disease, fibroadenomas, and breast benign hyperplasia are the leading causes of false-positive BSGI results [17]. Furthermore, malignant breast tumors drive local angiogenesis, and certain benign growths such as intraductal papillomas, inflammatory lesions, fibroadenomas, or adenopathies can mimic this activity and are associated with abundant blood supplies. Inflammatory lesions also exhibit features including irregular infiltration of the surrounding tissue. Weight et al. [18] found that BSGI was associated with a higher incidence of altered patient management (109/119) than was ultrasound (71/119), supporting the fact that BSGI achieves higher levels of positive predictive value and accuracy than does ultrasound. In this study, we detected 31 false-positive lesions via BSGI, with this rate being lower than the 47 false-positive lesions detected via ultrasound (Fig. 5).

MRI is accepted to be the optimal imaging modality for the diagnosis, staging, and monitoring of breast cancer in patients undergoing neoadjuvant chemotherapy (NAC) treatment in order to assess therapeutic responses [19]. Bilimoria et al. [20] found that breast MRIs are the most sensitive approach to detecting breast cancer, and that they have the potential to detect tumors that are not detectable upon physical examination, mammography, or ultrasound. However, breast MRIs can nonetheless yield high false-positive rates and appear to enlarge tumors, potentially leading women diagnosed via this approach to undergo an unnecessary mastectomy. MRI can also not be used regularly owing to its high costs. Hwang et al. [21] determined that preoperative MRI assessment was not sufficient to predict the odds of achieving negative margins for lumpectomy specimens, nor did such assessment reduce subsequent re-excision rates. As preoperative MRI is not associated with benefits associated with ipsilateral breast tumor recurrence (IBTR), there is no cause at present to recommend that preoperative MRI be integrated into the routine assessment of all women with newly diagnosed breast cancer.

Other imaging approaches such as quantitative shear-wave elastography (SWE) have also been employed to enhance breast cancer diagnostic accuracy. As malignant breast lesions are harder than healthy breast tissue, SWE can both quantify tissue hardness and offer information regarding tumors. Yoon et al. [22] analyzed 199 consecutive women and determined that SWE was associated with higher false-positive than false-negative rates, found size, depth, and breast thickness all having impacted these findings. Kim et al. [23] also reported in their study of 166 total masses (118 benign, 48 malignant) that false SWE features were more commonly detected in benign masses (53% vs. 8.2%).

Recent advances in digital x-ray system development have also facilitated the design of approaches such as contrast-enhanced spectral mammography (CESM) that can overcome many of the limitations of mammography and achieve diagnostic efficacy comparable to MRI. Xing et al. [19] found that CESM achieved higher specificity than MRI (89.5% vs 80.2%) and that the same was true for its positive predictive value (94.7% vs 90.5%). However, this approach is limited by the fact that it is unable to image the entire chest wall and axilla, requires compression, utilizes ionizing radiation, and requires that patients receive iodinated contrast injection. In a prospective two-center, multi-reader study, Fallenberg et al. [24] determined that MRI and CESM exhibited comparable diagnostic performance, but that CESM had higher specificity and lower sensitivity relative to MRI. Given that radiation dosing is always a pertinent concern, it is worth noting that combination CESM + MMG imaging delivers radiation doses higher than mammography alone. In addition, the invasiveness of this approach and the need for contrast injection make it unsuitable for breast cancer screening.

### Limitations

There are still several limitations to the utilization of BSGI as a means of evaluating breast lesions. For one, it is unable to image the entire chest wall and it offers poor sensitivity as a means of detecting axillary lymph nodes. In addition, BSGI results in patients being exposed to 6.29–9.44 mSv of radiation, and as such it should not be conducted indiscriminately [15, 25]. In addition, lower doses and longer acquisition times may be necessary for the evaluation of patients with suspicious lesions or dense breast tissue. Furthermore, given that this was a planar test, improper positioning has the potential to affect result accuracy [12, 26]. The advantages and disadvantages of BSGI, mammography, ultrasound, and MRI are shown in Table 3.

**Table 3:** Advantages and disadvantages of BSGI, mammography, ultrasound, and MRI

Approach	Amount of ionizing radiation	Costs	Total imaging time	Contraindication	Convenience
BSGI	6.29–9.44 mSv	Proper	20–30 min	None	No
MRI	None	Expensive	20–30 min	Yes	No
Mammography	0.5 mSv [27]	Inexpensive	5–10 min	None	Yes
Ultrasound	None	Inexpensive	5–10 min	None	Yes

## Conclusions

In summary, the results of this study demonstrate that, relative to ultrasound and mammography, BSGI is a highly sensitive and specific tool that can be used to reliably evaluate breast lesions. BSGI achieved sensitivity that was comparable to that of MRI when used as an auxiliary diagnostic imaging modality to ultrasound and mammography, suggesting that it can further enhance diagnostic efficacy in this context. As such, our findings highlight the value of the clinical application of BSGI as a means of differentiating between benign and malignant breast lesions with the goal of reducing the incidence of misdiagnosis and unnecessary surgery.

## Data Availability

The datasets used and/or analysed during the current study are available from the corresponding author on reasonable request. Anyone who is interested in the information should contact hbliu@zju.edu.cn.

## Abbreviations

BSGI: Breast specific gamma imaging
MRI: Magnetic resonance imaging
IDC: Invasive ductal carcinomas
DCIS: Ductal carcinomas in situ
LNR: Lesion-to-normal tissue ratio
CC: Craniocaudal
MLO: Mediolateral oblique
PPV: Positive predictive value
NPV: Negative predictive value
ER: Estrogen receptor
PET/CT: Positron emission tomography with computed tomography
IBTR: Ipsilateral breast tumor recurrence
NCCN: The National Comprehensive Cancer Network
SWE: Shear-wave elastography
CESM: Contrast-enhanced spectral mammography
